# Synthesis and Characterization of Ag/ZnO Nanoparticles for Bacteria Disinfection in Water

**DOI:** 10.3390/nano12101764

**Published:** 2022-05-22

**Authors:** Julia de O. Primo, Dienifer F. Horsth, Jamille de S. Correa, Arkaprava Das, Carla Bittencourt, Polona Umek, Ana Guilherme Buzanich, Martin Radtke, Kirill V. Yusenko, Cristina Zanette, Fauze J. Anaissi

**Affiliations:** 1 Departamento de Química, Universidade Estadual Do Centro-Oeste, Guarapuava 85040-080, Brazil; juliadeoliveira@unicentro.edu.br (J.d.O.P.); dhorsth@unicentro.edu.br (D.F.H.); jscorrea@unicentro.edu.br (J.d.S.C.); anaissi@unicentro.edu.br (F.J.A.); 2 Chimie des Interactions Plasma-Surface (ChIPS), Research Institute for Materials Science and Engineering, University of Mons, 7000 Mons, Belgium; arkaprava.das@umons.ac.be; 3 Solid State Physics Department, Jožef Stefan Institute, 1000 Ljubljana, Slovenia; polona.umek@ijs.si; 4 Federal Institute for Materials Research and Testing (BAM), 12205 Berlin, Germany; ana.buzanich@bam.de (A.G.B.); martin.radtke@helmholtz-berlin.de (M.R.); kirill.yusenko@bam.de (K.V.Y.); 5 Departamento de Engenharia de Alimentos, Universidade Estadual do Centro-Oeste, Guarapuava 85040-080, Brazil; czanette@unicentro.br

**Keywords:** zinc oxide, silver nanoparticles, water disinfection

## Abstract

In this study, two green synthesis routes were used for the synthesis of Ag/ZnO nanoparticles, using cassava starch as a simple and low-cost effective fuel and Aloe vera as a reducing and stabilizing agent. The Ag/ZnO nanoparticles were characterized and used for bacterial disinfection of lake water contaminated with *Escherichia coli* (*E. coli*). Characterization indicated the formation of a face-centered cubic structure of metallic silver nanoparticles with no insertion of Ag into the ZnO hexagonal wurtzite structure. Physicochemical and bacteriological analyses described in “Standard Methods for the Examination of Water and Wastewater” were used to evaluate the efficiency of the treatment. In comparison to pure ZnO, the synthesized Ag/ZnO nanoparticles showed high efficiencies against *Escherichia coli* (*E. coli*) and general coliforms present in the lake water. These pathogens were absent after treatment using Ag/ZnO nanoparticles. The results indicate that Ag/ZnO nanoparticles synthesized via green chemistry are a promising candidate for the treatment of wastewaters contaminated by bacteria, due to their facile preparation, low-cost synthesis, and disinfection efficiency.

## 1. Introduction

Water is a key resource for the existence of life on Earth; therefore, water access is considered one of the most critical humanitarian goals. Nevertheless, according to World Health Organization (WHO) [1] in 2020, only 50% of the population had access to safely managed drinking water, while the remaining 50% still lacked access to drinking water sources and safely managed services. The same source reports that, on a global scale, two billion people use drinking water sources contaminated with feces, and the microbial contamination of drinking water may transmit diseases such as diarrhea, cholera, dysentery, polio, and typhoid, which is estimated to cause 485,000 diarrheal deaths each year.

Drinking water legislation requires microbial, physical, and chemical water parameters to be met to ensure adequate public health conditions [2]. Knowing that, the bacterial contamination of water is a concern, as it causes diseases that can be life-threatening upon ingestion or exposure. Various traditional water disinfectant methods, along with chemical (chlorination, ozonation) and physical (ultraviolet radiation) methods, are effective, and among these, chlorine disinfection has been used to prevent infections for more than a century [3,4]. Although the disinfection methods currently used in drinking water treatment can effectively control microbial pathogens, effective disinfection results in the formation of harmful disinfection byproducts (DBPs). For example, the water industry uses free chlorine, chloramines, and ozone for disinfection; these products can react with various components presents in water to form DBPs [3,5,6].

Given the limitations of conventional disinfection methods, advances in nanotechnology have the potential to generate novel treatment capabilities that may allow economic utilization of unconventional water sources to expand the water supply [6,7]. Depending on their chemical composition, nanomaterials are promising alternatives offering leapfrogging opportunities to develop next-generation water supply systems due to their large specific surface areas and high reactivity, which make them excellent adsorbents, catalysts, sensors, and as recent studies have shown, antimicrobial agents. For example, silver nanoparticles are an antibacterial nanomaterial with strong oxidant properties that are relatively inert in water [5,7].

The effective antibacterial activity of silver (Ag) nanoparticles (NPs) for a wide spectrum of bacteria, viruses, protozoa, algae, and yeasts opens up opportunities for their use in water disinfection [8]. Ag NPs have been widely used against Gram-negative bacteria due to their higher activity against them. However, Ag NPs in contact with the environment during water treatment may pose a health risk, as the level of toxicity to the end user is not well known. For water disinfection, Ag NPs can be combined with particles of inorganic oxides to prevent the silver leaching into the water due the formation of water-soluble species. Among the inorganic particles that have been tested against several pathogenic and non-pathogenic bacteria, zinc oxide (ZnO) has been reported to have high antimicrobial activity [3,9,10]. ZnO particles are also chemically stable, have large surface areas, and have strong antibacterial properties, even in very small quantities [4,11]; and most importantly ZnO particles have been reported to be non-toxic to human cells [12]. Compared to organic materials, inorganic materials such as ZnO have greater durability, selectivity, and heat resistance [6].

Both Ag and ZnO particles present satisfactory antibacterial results; however, when used as individual components, they tend to agglomerate, which reduces their effectiveness [3]. The use of noble metals such as silver (Ag) to modify the ZnO surface can increase the chemical stability and antibacterial properties [13]. In recent years, methods for obtaining nano-Ag/ZnO have been reported for different applications, such as photocatalysis [14,15], controlled drug release [16], and antimicrobial activity [17].

In this study, ZnO particles decorated with silver nanoparticles were synthesized via two low-toxicity synthesis routes: route (I) is a one-step synthesis that uses starch as a complexing gelling and combustion power, and route (II) has two steps synthesis. In the first step pure ZnO nanoparticles are synthesized, and in the second step Ag decoration on their surface is performed using Aloe vera leaf extract as the reducing agent for Ag^+^. The samples were investigated using X-ray Diffraction (XRD), Raman Spectroscopy, X-ray photoelectron spectroscopy XPS, X-ray Absorption Near Edge Structure spectroscopy (XANES), and Extended X-Ray Absorption Fine Structure spectroscopy(EXAFS). XPS was used to evaluate the near surface chemistry of the nanoparticles because the X-ray absorption (XAS) measurements were performed in transmission mode; therefore, they may not indicate the presence of very thin oxide layers on the particle’s surface that can be detected by XPS. The morphology of the samples was determined by field-emission scanning electron microscopy (SEM) and transmission electron microscopy (TEM). The atomic distances from the Zn and Ag atoms were calculated. The antimicrobial activity of the synthesized Ag/ZnO nanoparticles was tested using contaminated water from a lake with Gram-negative *Escherichia coli*, to evaluate the efficacy of these nanomaterials when applied for disinfection during water treatment.

## 2. Materials and Methods

### 2.1. Materials

All reagents used in the synthesis of the samples were of analytical grade and were used as received. The inorganic salts used were zinc nitrate hexahydrate (Zn(NO_3_)_2_⋅6H_2_O), 98%, NEON, Suzano, Brazil) and silver nitrate (AgNO_3_, 99.8%, Quimex, Cotia, Brazil. All solutions were prepared with deionized water. Natural Cassava starch in the form of colloidal suspension was used as fuel [18]. Aloe vera leaves were harvested in the São José region of Parana-Brazil (Parana, Brazil).

### 2.2. Synthesis of ZnO Using Cassava Starch: Pure and Ag/ZnO (Route I)

Pure and Ag/ZnO were obtained by the gelatinization method. First a white matrix was prepared from 200 g of starch in 1000 g of water; second, the Zn(NO_3_)_2_⋅6H_2_O in the proportion of 98% to 2% (*w*/*w*) was added. This suspension (zinc/starch) was kept under stirring at room temperature (RT) until complete dissolution of the emulsion. After 60 min of mechanical stirring (600 rpm), the suspension was calcined in a muffle furnace at 750 °C for 1 h to obtain the pure ZnO [18].

**Figure 1:**
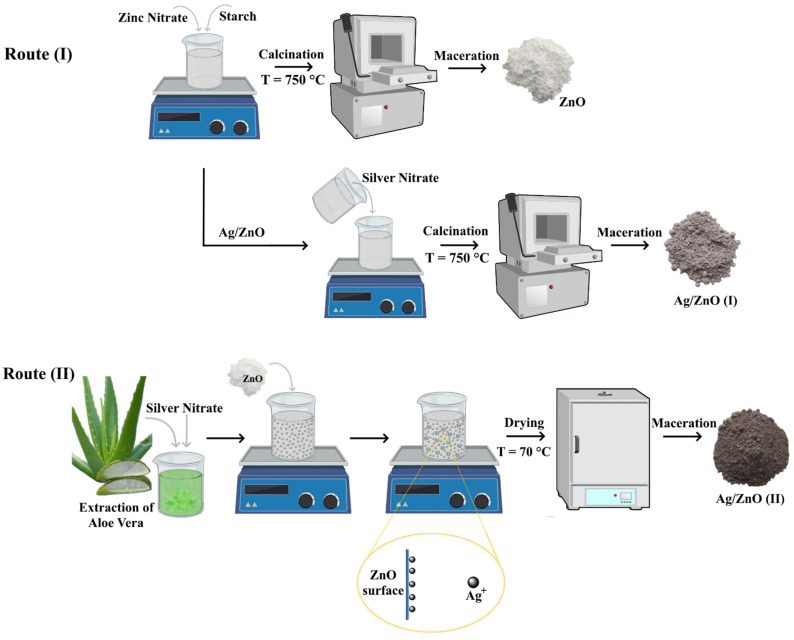
Schematic diagram of the synthesis routes used.

The Ag/ZnO nanoparticles were prepared by adding silver (I) ions (10% *w*/*w*) to the zinc solution containing starch and zinc nitrate hexahydrate. Then, 9.28 g of Zn(NO_3_)_2_⋅6H_2_O and 0.32 g of AgNO_3_ were added to the starch matrix during the formation of the emulsion. After 60 min under of mechanical stirring (600 rpm), the suspension was calcined in a muffle furnace at 750 °C for 1 h (Figure 1).

### 2.3. Synthesis of Ag/Zinc Oxide (Route II)

To obtain the extract of Aloe gel, 5 g of Aloe vera leaves were thoroughly washed, then the leaves were finely cut and boiled in 100 mL of deionized water. Next, the leaves and mucilaginous gel were crushed using a pistil and a ceramic mortar to obtain the complete extract. Finally, the solution was washed and filtered, and the resulting Aloe vera gel broth extract was stored under refrigeration (2 °C) for further experiments.

For the synthesis of silver nanoparticles, 0.5 g of AgNO_3_ was added to 50 mL of Aloe vera extract, related to (10% *w*/*w*) the ZnO. The solution was kept under constant magnetic stirring for 12 h. Afterwards, 3 g of ZnO obtained in route (I) was added to the solution and kept under constant magnetic stirring for 4 h. The obtained product was centrifuged, washed with water and ethanol, and dried at 70 °C in an oven for 12 h (Figure 1). The obtained material was labeled Ag/ZnO (II).

### 2.4. Characterization Techniques

The crystalline phases were identified by powder X-ray diffraction (XRD) performed on a Bruker model D2 Phaser with Cu Kα radiation (***λ*** = 1.5418 Å), with scans in 2*θ* from 20° to 70° and step rate of 0.2 °/sec. The crystallite size (***D***) was estimated by the Scherrer equation [19]:

(1) $$D=\frac{k\lambda }{\beta \cos\left(\theta \right)}$$

*k* is the form factor, *λ* is the wavelength of incident X-rays, *β* is the full width at half-maxima (FWHM) of the maximum intensity peak, and *θ* is the Bragg angle of the maximum intensity peak.

Raman spectra were recorded using a Micro-Raman system (Senterra Bruker Optik GmbH, Massachusetts, USA), ***λ*** = 532 nm, laser power 5 mW, time 10 s, resolution 4 cm^−1^. The morphology of the samples was examined using a field-emission scanning electron microscope (JSM − 7600F, JEOL, Tokyo, Japan) and a transmission electron microscope (JEM − 1011, JEOL, Tokyo, Japan). For SEM analysis, specimens were prepared by dispersing the powder samples in a small amount of deionized water, and a drop of the dispersion was deposited on a polished surface of an Al sample holder. Prior to the SEM investigation, an ca. 4 nm thick carbon layer was deposited on the specimens to reduce the charging effect. Energy dispersive X-ray (EDX) mapping and line analyses were preformed using SEM (Verios 4G HP, ThermoFischer, Waltham, MA, USA).

Specimens for TEM investigation were prepared by dispersing the prepared samples of NPs ultrasonically in methanol, and a drop of the dispersed solution was deposited onto a lacy carbon film supported by a copper grid.

The oxidation state and composition of the chemical elements at the nanoparticle surface were evaluated by X-ray photoelectron spectroscopy (XPS) (Versaprobe PHI 5000, from Physical Electronics, Chanhassen, MN, USA), equipped with a monochromatic Al Kα X-ray source. The XPS spectra were collected at a take-off angle of 45° with respect to the electron energy analyzer, and the spot size was 200 μm. Pass energy (PE) of 20 eV was used for the high-energy resolution spectra (Zn 2p, O 1s, Ag 3d, and C 1s). The spectra were analyzed using the CASA-XPS software, and the binding energy of the XPS spectra was calibrated using the C 1s peak at 284.6 eV [20]. Multipack version 9.8 software (ULVAC-PHI, 2017, Chigasaki, Japan) was used to evaluate the relative composition of the elements.

In order to investigate the local structural properties of Ag NPs in ZnO, the extended X-ray absorption fine structure (EXAFS) spectra were recorded with standard transmission setup at the Zn K edge (9.6586 keV) and Ag K edge (25.5140 keV) at BAMline at the Helmholtz–Zentrum Berlin (BESSY II, Berlin, Germany) [21]. The X-ray beam was monochromatized with a Si(111) double crystal monochromator (ΔE/E = 2 × 10^−4^). The energy was scanned in 10 eV steps until 20 eV before the edge, followed by 0.5 eV steps around the edge until 100 eV, and then 1.5 eV steps until 200 eV. From then on, acquisition in 0.04 ${Å}^{-1}$ equidistant *k*-steps was carried out until *k* = 16 ${Å}^{-1}$. Samples in powder form were mixed with hexagonal BN powder to get enough transmission and pressed between two Kapton foils to get 1 mm thick pellets. All measurements were performed with standard metal foil, i.e., Ag and Zn foil, for calibration, and repeated twice to check the reproducibility of the Ag K and Zn K-edge spectra. Initial data processing with background removal and Fourier transformation of EXAFS oscillation was performed with Athena software of the IFEFFIT package. Simulation of EXAFS spectra was accomplished with FEFF and fitted in Artemis software of the same package [22].

### 2.5. Water Disinfection

For the study, samples of contaminated water were collected during February 2022 from a lake located on the UNICENTRO, Campus Cedeteg, in the city of Guarapuava, Parana, Brazil. The samples were prepared in 100 mL sterile plastic flasks containing sodium thiosulfate pellets that are used for neutralization of chlorine present in the lake water.

#### 2.5.1. Preliminary Tests

The amount of the synthesized nanoparticles required for the disinfection process was optimized by testing different concentrations of Ag/ZnO particles. The disinfection process consisted of direct contact between the contaminated water (100 mL) and the prepared ZnO and Ag/ZnO samples (0.25, 0.5 and 1 g) for 15 min under constant agitation (300 rpm) at RT. Subsequently, filtration was carried out on filter paper, with a few seconds waiting time for the sample to settle in the filter. The treated water was collected in a sealed and sterilized bottle for microbiological and physical-chemical analysis at the Water Analyses Laboratory UNICENTRO.

#### 2.5.2. Water Disinfection Ability of Nanoparticles

Leveraging the optimization results, the water disinfection test was performed according to Section 2.5.1. Physicochemical and microbiological analyses were performed on the contaminated water and after treatment with the particles. The physicochemical and bacteriological analyses were carried out according to the methodologies described in “Standard Methods for the Examination of Water and Wastewater” [23]. The evaluated parameters were total coliforms (TC), pH, turbidity, and *Escherichia coli*. The methods used for analysis of these parameters are shown in Table 1.

## 3. Results and Discussion

### 3.1. XRD

**Figure 2:**
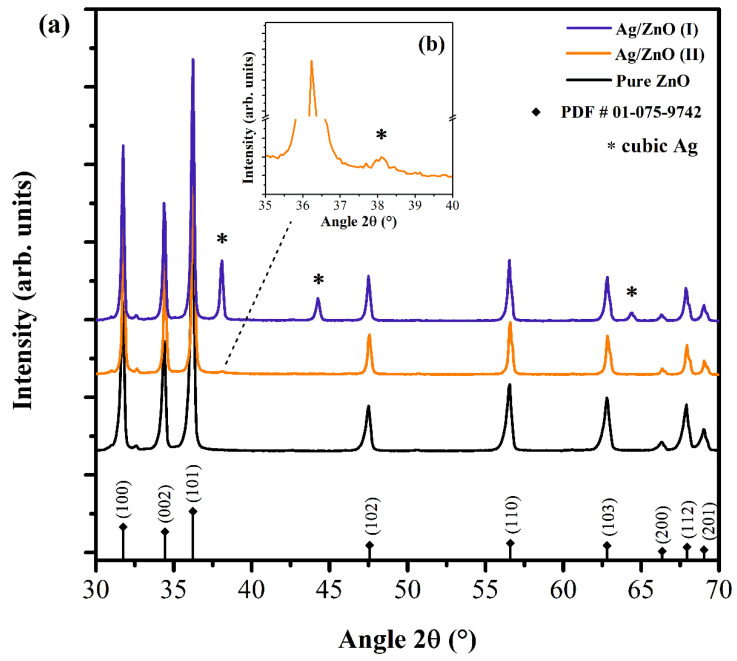
(**a**) XRD patterns of ZnO and Ag/ZnO prepared by route I and II; (**b**) the amplified XRD pattern in the range of 2θ = 35 – 40 for the sample Ag/ZnO (II).

The crystal structures of ZnO and Ag/ZnO samples were determined using X-ray diffraction (XRD). The diffraction patterns of the three samples (Figure 2) have peaks characteristic of the hexagonal wurtzite structure of ZnO (ICDD card number 01-075-9742). According to some prior reports, the incorporation of Ag ions into the ZnO lattice can be substituted for Zn^2+^ or as an interstitial atom [24,25]. In the case of silver replacing Zn^2+^, a corresponding peak shift would be expected in the XRD; however, for our samples, shifts were not observed, indicating that there was no substitutional doping. For Ag/ZnO (II), substitutional doping was not expected because the ZnO particles were already formed when Ag^+^ were added.

Additional diffraction peaks at 2*θ* = 38.1° (111), 44.2° (200), and 64.3° (311) are shown in the diffractogram of Ag/ZnO (I), corresponding to (111), (200), and (220) planes of the face-centered cubic (fcc) structure of Ag (ICDD card number 04-0783) [13,26,27]. For the sample obtained by route II, only a low intensity peak centered at 38.1° can be observed (Figure 2b). The absence or low intensity of other peaks characteristic of Ag indicates a low concentration and/or small Ag NPs [24,28,29]. This peak alone may be associated with the Ag fcc structure or Ag_2_O. Further, EXAFS analysis was performed to determine the local structure.

The crystallite sizes of the samples were calculated from the X-ray line broadening using Scherrer’s equation [19]. The average calculated values were 23.4 nm for pure ZnO, 32.5 nm for Ag/ZnO (I), and 33.9 nm for Ag/ZnO (II). This increase in crystallite size can be attributed to the Ag nanoparticles anchored to the surface of ZnO [14].

### 3.2. Raman Spectroscopy

**Figure 3:**
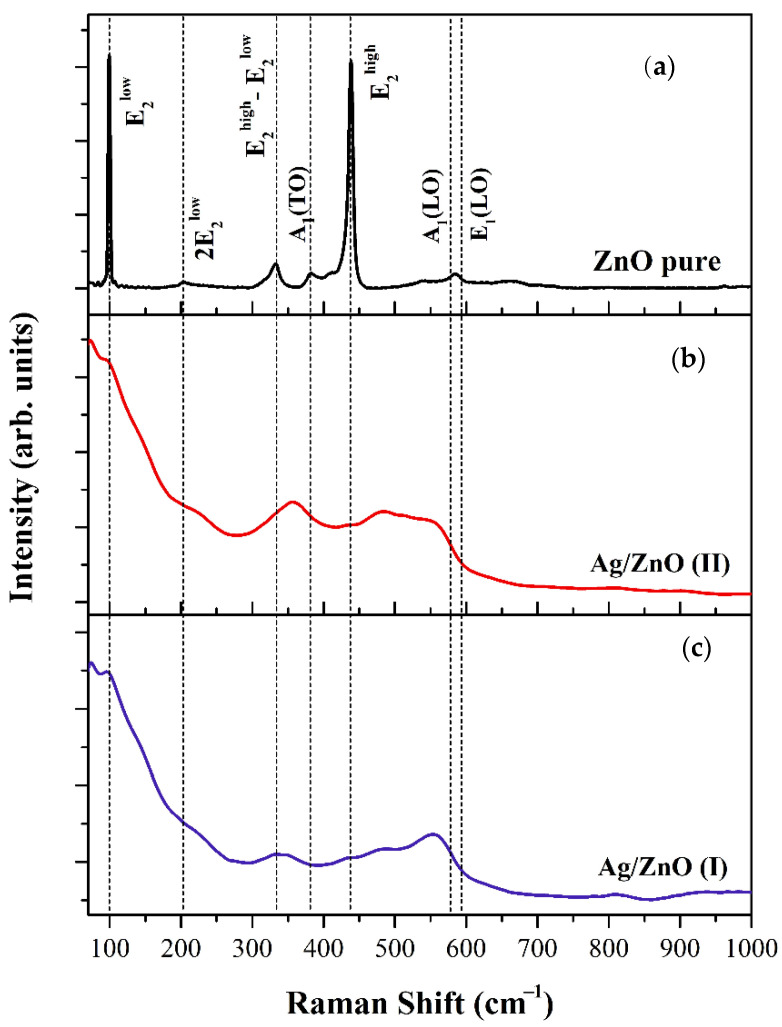
Raman spectra of synthesized (**a**) pure ZnO, (**b**) Ag/ZnO (I), and (**c**) Ag/ZnO (II) using starch and Aloe Vera Barbadensis miller extract.

In order to investigate the ZnO defects and chemical bonding at the Ag/ZnO interface, Raman spectra of all samples were measured (Figure 3). The Raman spectrum of the pure ZnO sample (Figure 3a) consisted of four predominant bands located at about 99, 380, 437, and 573 cm^−1^, which correspond to the fundamental phonon modes of ZnO wurtzite structure [18], in accordance with the XRD results. The Ag/ZnO spectra (Figure 3b,c) show characteristic peaks centered at 99 and 435 cm^−1^, which can be assigned the fundamental phonon modes E_2_^low^ and E_2_^high^ of hexagonal wurtzite ZnO, respectively [30,31]. The A_1_(LO) polar branches appeared at about 555 cm^−1^ for the samples decorated with Ag. Due to the deposition of Ag on ZnO, this mode was broadened and shifted toward lower energy. Such a shift and broadening of A_1_(LO) phonon mode can be attributed to the scattering contributions with the phonons away from the center of Brillouin zone [32]. The A_1_(LO) phonon mode is commonly assigned to the oxygen vacancies (V_o_) [32,33,34], zinc interstitial (Zn_i_) defects in ZnO [31,34], or defect complexes of combined V_o_ and Zn_i_ in the host lattice [34]. Additionally, a broad Raman peak positioned at about 483 cm^−1^ only appeared in the spectra of the samples containing Ag, which was assigned as the interfacial surface phonon mode in the literature [35,36]. This phonon mode was also observed for ZnO loaded with cobalt [36], and thus could not be a local vibrational mode associated with Ag cations.

### 3.3. SEM

**Figure 4:**
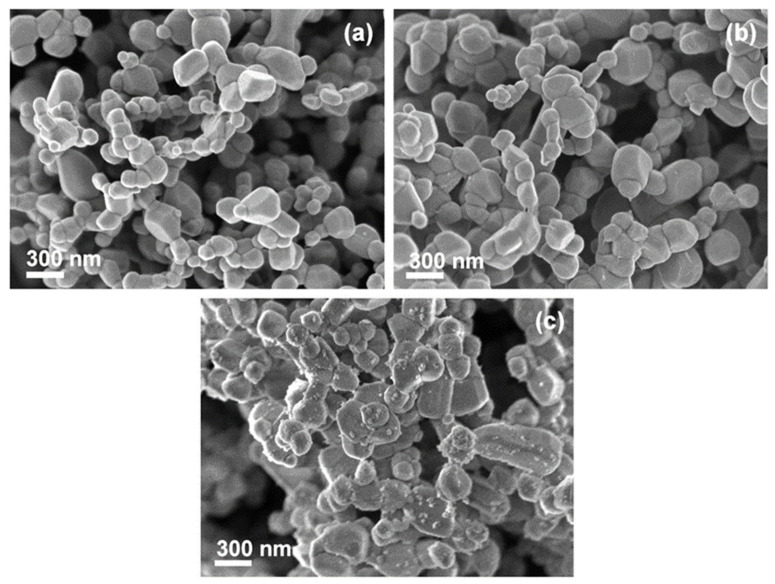
SEM images of pure ZnO (**a**), Ag/ZnO (I) (**b**), and Ag/ZnO (II) (**c**).

An investigation with SEM was used to assess the morphology and size of ZnO particles (Figure 4). The particles in all three samples had no particular shape, and their size was in the range of 100 to 350 nm. A comparison of the SEM images shown in Figure 4a,c with that in Figure 4b also revealed that the presence of Ag^+^ in the reaction mixture of Ag/ZnO (I) had no effect on the morphology and size of the ZnO particles.

To distinguish ZnO and Ag particles and to determine the Ag NP density distribution, the Ag-loaded ZnO samples were visualized with a back scattered electron (BSE) mode of SEM (BSE-SEM). First, the particles in bright contrast BSE-SEM images were identified as Ag NPs (Figures S1 and S2). A comparison of BSE-SEM images of both Ag/ZnO samples (Figure S3, see Supplementary Materials) revealed that: (i) the distribution of Ag NPs was denser in Ag/ZnO (II) and (ii) the Ag NPs in Ag/ZnO (I) were bigger (up to 300 nm) (Figure S1).

### 3.4. TEM

**Figure 5:**
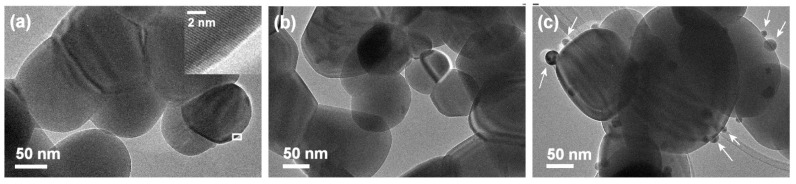
TEM images of (**a**) ZnO pure, (**b**) Ag/ZnO (I), and (**c**) Ag/ZnO (II). The white rectangle in the (**a**) defines the area of the inset. Arrows in the image (**c**) point toward Ag nanoparticles.

TEM was used to further examine the ZnO and Ag particles and determine the size of Ag NPs in Ag/ZnO (II). No amorphous layer around ZnO particles was observed (Figure 5 and Figure S4), indicating that during calcination, organic species were removed successfully. ZnO particles were crystalline (see inset of Figure 5a). The average measured d-spacing of 0.26 nm well corresponds with the (002) planes of the hexagonal wurtzite structure of ZnO (ICDD card number 01-075-9742) [37], and is in accordance with the results of Raman and XRD analyses. In Figure 5c, besides the presence of larger ZnO particles, smaller particles with diameters from 10 to 40 nm can be observed (arrows in Figure 5c are pointing to these particles). The measured inter-planner lattice spacing was 0.25 nm (Figure S4) and agrees well with (111) planes of fcc Ag.

### 3.5. XPS

**Figure 6:**
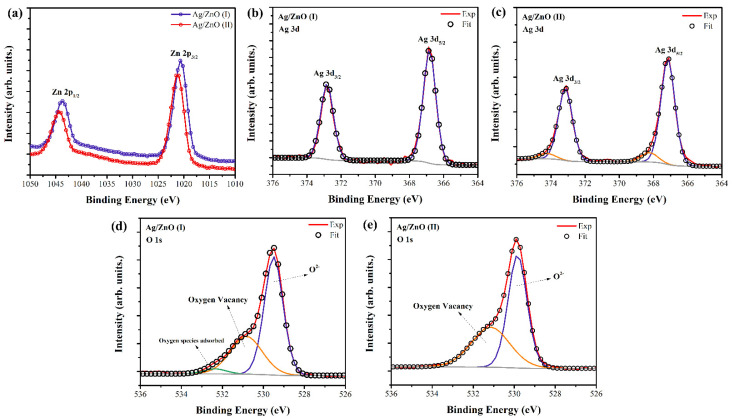
XPS spectra of Ag/ZnO particles obtained by two different synthesis routes. High-resolution regional XPS spectra of (**a**) Zn (2p), (**b**,**c**) Ag (3d), and (**d**,**e**) O (1s).

The chemical bonding states of Ag decorated ZnO samples were evaluated by analyzing the XPS spectra (Figure 6). The core-level XPS spectra of Zn (2p), Ag (3d), and O (1s) are shown in Figure 6a–c. In Figure 6a, the doublet peak with components at ~1021.0 and ~1044.0 eV for both Ag/ZnO samples were assigned to Zn 2p_3/2_ and Zn 2p_1/2_, respectively. These values agree with the binding energies of Zn 2p3/2 and Zn 2p1/2 in stoichiometric ZnO, which is attributed to Zn^2+^ state oxidation [38,39].

Figure 6b shows the spectrum recorded in the region of the Ag 3d core level. For the sample Ag/ZnO (I), the components of the Ag 3d core levels are centered at 366.8 eV (Ag 3d_5/2_) and 372.8 eV (Ag 3d_3/2_). The spin-orbit splitting of 6 eV indicates that Ag atoms were mainly in the metallic state Ag^0^ [39]. The Ag 3d core-level binding energy in the Ag/ZnO (I) sample was shifted to the lower binding energy compared to the corresponding value of the pure metallic Ag (Ag 3d_5/2_ is about 368.2 eV). This was associated with the interaction between Ag NPs on the surfaces of ZnO nanocrystals, which led to the adjustment of Fermi level [39,40]. These results are in good agreement with the presence of the fcc metallic Ag crystal structure obtained by XRD. As can be seen, the Ag 3d_5/2_ spectrum of the Ag/ZnO (II) sample was fitted by two components centered at 367.1 and 368.2 eV, corresponding to Ag_2_O and Ag^0^ electronic states, respectively [37,38,39,41]

The O (1s) core-level spectra illustrated in Figure 6d were fitted with three components centered at 529.5, 530.8, and 532.6 eV for sample (I), and two components centered on 529.8 and 531.2 eV for sample (II) (Figure 6f). The peak with the lower binding energy (~529.0 eV) was attributed to O^2−^ ions participating in the Zn–O bonding in the wurtzite structure of the hexagonal ZnO [42,43]. The component centered at ~531.0 eV is associated with photoelectrons emitted from O^2−^ ions in oxygen-deficient regions in the matrix of ZnO [42]. As shown in Figure 6c, the sample obtained in synthesis route (I) had one more component at 532.5 eV, which was attributed to oxygen species chemisorbed on the surface of the Ag/ZnO [44]. The adsorbed oxygen species in Ag/ZnO (I) may affect the physical and chemical properties of the material.

### 3.6. EXAFS

The local environments of the Ag and Zn atoms in the samples were probed by EXAFS spectra of Ag K edge and Zn K edge. The EXAFS oscillations occur due to the interference between outgoing and backscattered photoelectron wave. The scattering signal *χ*(*E*) is defined as follows [45]:

(2) $$\chi \left(E\right)=\frac{\mu \left(E\right)-\mu_{0}\left(E\right)}{\Delta \mu_{0\;}\left(E_{0}\right)}$$

$E_{0}$ is the tabulated energy of an absorption edge, $\Delta \mu_{0\;}\left(E_{0}\right)$ is step or edge jump between pre- and post-edge lines, $\mu_{0\;}\left(E_{0}\right)$ is the mass absorption coefficient of the isolated atom, and μ(E) is the mass absorption coefficient of the element in the sample. For EXAFS interpretation, it is convenient to have the data in *k*-space, which follows the mathematical relation.

(3) $$k=\sqrt{\frac{2m\left(E-E_{0}\right)}{\hbar^{2}}}\;$$

m represents the electron mass in the Equation (3). In the Fourier transformed plot, the actual distance of the oscillation from the photo-absorbing atom is plotted. The background removal, normalization, and Fourier transformation of the $k^{2}$ weighted χ(k) spectra were performed with Athena software from the IFEFFIT package [22]. Simulation of the Ag K edge and Zn K edge was performed with FEFF within Artemis software. From the XRD pattern in Figure 1, we already had an idea about the existing phases. Therefore, considering the concerned crystallographic information file (CIF), the different scattering paths were obtained mainly for the first coordination shell around the central Ag and Zn atoms. With the cubic Ag and hexagonal wurtzite CIF file, the Ag–Ag, Zn–O, and Zn–Zn paths were considered for simulation of Ag K and Zn K edges, respectively. Debye–Waller factor (σ^2^), nearest neighbor coordination number (CN), and bond distance (c) were considered as variable parameters during simulations. We calculated the amplitude reduction factor ($s_{0}^{2})$ from the simulation of standard metallic foil’s (Ag and Zn) EXAFS spectrum and used the same value for simulating the EXAFS profile for Ag-K and Zn-K edges of the doped oxides samples.

**Figure 7:**
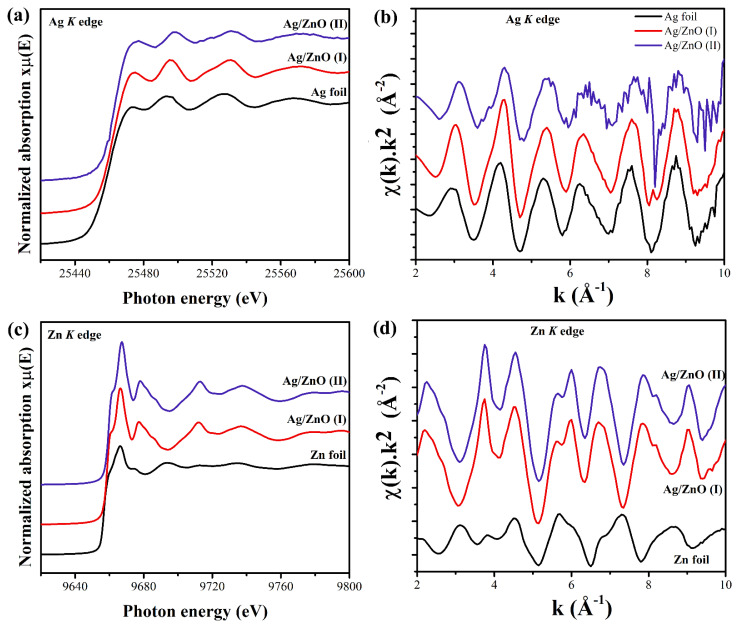
Ag loaded ZnO NPs. (**a**,**c**) Normalized XANES data for Ag K edge and Zn K edge. (**b**,**d**) k^2^-weighted *χ*(*k*) spectra for Ag K edge and Zn K edge. XANES and *χ*(*k*) were both shifted vertically and stacked for observational clarity.

**Figure 8:**
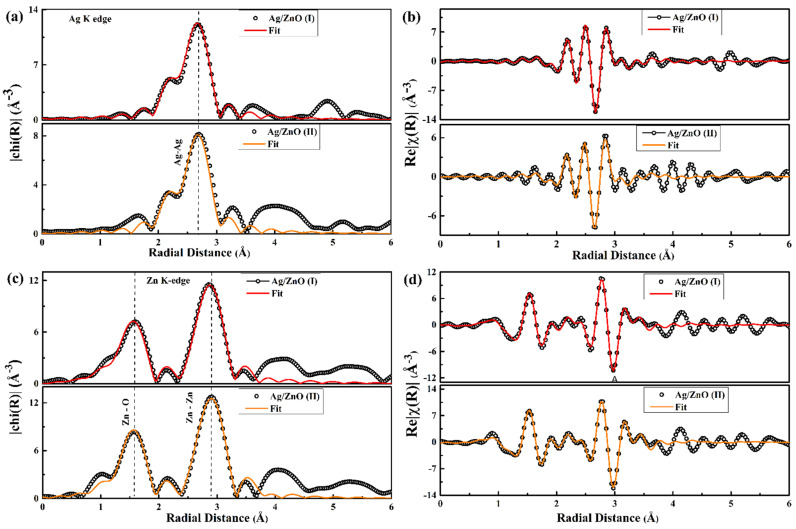
(**a**,**c**) Magnitude and (**b**,**d**) real component of χ(R) for Ag/ZnO (I) and Ag/ZnO (II) samples.

Figure 7a,c shows the normalized XANES spectra for the Ag K edge and Zn K edge. Figure 7b,d portrays the $\chi \left(k\right).k^{2}$ EXAFS spectra for Ag K edge and Zn K edge. In Figure 7a, no changes are visible in the intensity or scattering features of the Ag K edge for both samples as compared to the Ag foil, which indicates the same local environment around the photo-absorbing Ag atom, and proves the presence of a metallic Ag phase. There was no change observed in the Zn K edge in XANES region for both samples (Figure 7c). The fitting of the Ag K edge in Figure 8a with the cubic Ag cif file indicates that there is no variation in Ag in coordination number between Ag/ZnO(I) and Ag/ZnO(II) samples. The same is the case for the Zn K edge in Figure 8c, showing that coordination number does not vary and no charge transfer took place due to any kind of cationic substitution between Ag and Zn. The Zn K edge fitting was performed with the hexagonal wurtzite cif file. The ionic radii of Ag^2+^ (0.94 Å) and Zn^2+^ (0.60 Å) are significantly different [46]. The change in Gibbs’ free energy with cationic substitution might be higher as compared to the bond formation energy of Ag–Ag bonding. Therefore, it is easier for Ag to make a bond with another nearby Ag atom rather than cationic substitution, resulting in a metallic Ag phase in both of the samples. The amplitude reduction factor ($s_{0}^{2}$) was calculated with a standard metallic foil of Ag K edge and Zn K edge. For the Ag K edge and Zn K edge, the $s_{0}^{2}$ values were found to be 0.7 and 1, respectively, which were kept fixed for all fits. Figure 8a–d portrays the EXAFS fitting for magnitude and the real part of the χ(R) spectra for Ag K and Zn K edges. From this simulation, we calculated the CN, R, and σ^2^ values, i.e., for Ag–Ag, Zn–O, and Zn–Zn single scattering paths. The k-range for Fourier transformation was 3 to 11.5 ${Å}^{-1}$ during the simulation of the Zn K edge. The same for the Ag K edge was 3 to 9.5 ${Å}^{-1}$. The R ranges were 1.85–3.15 Å and 1–3.65 Å for Ag and Zn fits, respectively.

Using the fcc Ag cif file, the EXAFS fitting of experimental Ag K edges for Ag/ZnO (I) and Ag/ZnO(II) samples is shown in Figure 8a. For the Ag/ZnO (I) sample, the Ag atom was coordinated with the 11.9 nearest Ag atoms with a bond length of 2.87 (0) Å. The standard fcc metallic Ag has fcc structure and 12 as the first coordination number (CN). This implies high crystallinity and no ionic substitution for Ag unit cells in the Ag/ZnO (I) sample, as this had the same CN as the standard fcc Ag unit cell. However, for the Ag/ZnO (II) sample, the CN was reduced to 8.9. However, the bond lengths (2.86 (0) Å) were the same. The reduced CN indicates the presence of Ag vacancies in the first coordination shell. The presence of such vacancies might be the reason behind tiny increment in σ^2^ values from 0.008 (0) ${Å}^{2}$ to 0.009 (0) ${Å}^{2}$. The presence of cationic interstitials or vacancies increases the σ^2^ value [47]. Therefore, both samples, Ag/ZnO (I) and Ag/ZnO (II), indicate the presence of metallic Ag with both of the synthesis methods.

The real part of χ(R) R spectra for the Zn K edge in Figure 8c indicates two major peaks corresponding to the nearest Zn–O coordination and second nearest Zn–Zn coordination. The modelling for the Zn K edge was performed with the hexagonal wurtzite ZnO cif file. The R values for Zn–O and Zn–Zn do not indicate any significant changes in Ag/ZnO (I) or Ag/ZnO (II) samples with different synthesis routes. The CN values for Zn–O coordination were 2.9 (4) and 2.9 (3) for Ag/ZnO (I) and Ag/ZnO (II) samples, which are different from the ideal ZnO lattice values. In ideal wurtzite ZnO cif file Zn–O CN is 4. The reduced Zn–O CN suggests the presence of V_o_ in both of the samples. These fitting results regarding the presence of V_o_ are in complete agreement with our retrospective O 1s edge XPS analysis. The second nearest neighbor, i.e., Zn–Zn CN, was 11.9 for both samples, alongside no noteworthy change in the Zn–Zn R value. The σ^2^ values also remained the same for Zn–Zn coordination in both samples. All results from fitting of the Ag K edge with fcc Ag structure and the Zn K edge with wurtzite ZnO structure are mentioned in Table 2.

### 3.7. Water Disinfection

For optimization of the sample mass used in the disinfection of the water, preliminary tests were carried out using 0.25, 0.5, and 1.0 g of the particles. Considering both the microbiological and physicochemical parameters, the best performance was obtained using 0.5 g of sample because it met the requirements for absence of total coliforms and *Escherichia Coli* (Table 3) and kept the disinfected water sample within the pH and turbidity limits accepted by the standard Standard Methods For The Examination of Water and Wastewater [23]. For the larger (1 g) and smaller (0.25 g) masses, the pH and turbidity of the water after disinfection were not satisfactory.

**Figure 9:**
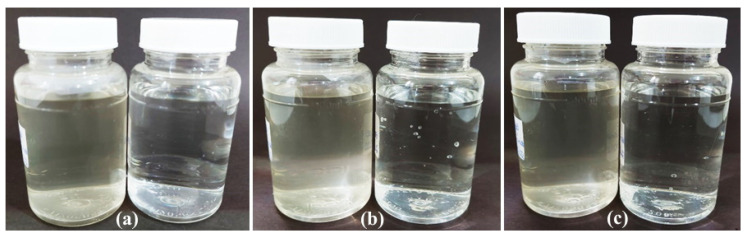
Pictures of the wastewater before and after disinfection tests using: (**a**) pure ZnO, (**b**) Ag/ZnO (I), and (**c**) Ag/ZnO (II).

The results in Table 3 are in accordance with the Standard Methods For The Examination of Water and Wastewater—23th Edition—SMEWW [23]. According to this method, the microbiological parameter limit for total coliforms and *Escherichia coli* in the water is “<1 or absence MPN/100 mL,” and as for the physicochemical parameters, the pH must be between 5.0 to 9.0 and the turbidity between null to 5.0. The effluent showed the count of > 2419.6 MPN/100 mL of total coliforms and 59.4 MPN/100 mL for *E. coli*. Figure 9 shows the effluent before and after treatment using ZnO and Ag/ZnO samples. After treatment with the pure ZnO sample, there was not total elimination of total coliforms or *E. coli* from the water. After disinfection with the pure ZnO, the pH increased to 8.2 and turbidity decreased to 95% of the original value (Table 3). After using the Ag/ZnO nanoparticles, no significant change in pH was observed, and the turbidity decreased to 93% or 90% after disinfection with Ag/ZnO (I) or Ag/ZnO (II), respectively (Table 3). It is known that various factors compromise the bacterial efficiency, such as turbidity, chloride, presence of organic matter, and pH [48]; however, the parameters assessed (pH and turbidity) did not affect the efficacy of silver-containing materials relative to total coliforms and *E. coli*, as the samples Ag/ZnO (I) and Ag/ZnO-Ag (II), physicochemical parameters, and microbiological parameters were within the limits permitted by the standard [23]. In this context, the absence of *E. coli* (<1 MPN/100 mL) indicated that the water after disinfection with Ag/ZnO nanoparticles did not present a sanitation risk [49].

According to the results, ZnO exhibited higher antibacterial efficiency when decorated with Ag NPs, suggesting that Ag NPs promote the antibacterial efficiency of ZnO, and this can be explained by the widely known bacterial effect of Ag nanoparticles [50,51]. As shown in the TEM analyses, the Ag/ZnO (I) was composed of Ag NPs at the nanometer scale. Several works report on the sizes of Ag particles in their antimicrobial properties against *E. coli* [52,53]. These results indicated that Ag/ZnO exhibited strong antimicrobial activity against *E. coli* compared to pure ZnO.

The mechanisms of antibacterial activity of ZnO and Ag NPs have not yet been clearly elucidated. However, some hypotheses have been proposed for the antimicrobial property of ZnO, such as the generation of reactive oxygen species (ROS) on the surfaces of the particles, release of zinc ions, membrane damage, and alteration of protein and nucleic acid functions [51,54,55,56]. On the other hand, AgNPs’ Ag^+^ ions may be attached to the negatively charged proteins and nucleic acids, leading to structural changes within the cell wall and membrane [57], Ag NPs penetrating inside the bacterial cell, resulting in DNA damage, and adhesion of nanoparticles to the surface altering the membrane properties [5,57]; and dissolution of AgNPs may release Ag^+^ ions [5,50,58], which may be disruptive to the cell walls. Considering the efficiency in disinfection performance, these materials obtained by green syntheses are potential alternative disinfectant agents to chlorine and its derivatives, which despite being widely used, has harmful impacts on human health [59].

#### Characterization of the Ag/ZnO Samples after Water Disinfection

XPS analyses of Ag/ZnO samples were carried out before and after their use in the disinfection treatment to investigate if the Ag and Zn (at%) concentrations varied, and we then evaluated the leaching of silver ions in water. Table 4 summarizes the results. Taking into consideration the error in the evaluation, we can observe that the relative amount of Ag did not vary significantly.

## 4. Conclusions

Pure ZnO and Ag-loaded ZnO were successfully synthesized by an eco-friendly method using Cassava starch and Aloe vera leaves. Aloe vera was used as a reducing agent and the starch in metal complexing and fuel for the reaction. The XRD results confirm that the decoration with silver did not alter the structural properties of pure ZnO that remained pure hexagonal wurtzite. The presence of Ag NPs on the surface of ZnO particles was confirmed by TEM and XPS. XANES and EXAFS denote the presence of metallic Ag NPs with both synthesis methods.

The synthesized pure ZnO and Ag-loaded ZnO through different methods were applied to wastewater disinfection. The Ag/ZnO suspensions showed a bactericidal effect against *E. coli* and total coliforms, exhibiting potential to be also used as an effective prophylactic agent for the disinfection of wastewater.

## Figures and Tables

**Table 1:** Methods used for analysis of the parameters evaluated in the present study.

Parameter Analyzed	Method
Total coliforms	SMEWW 9223 B–Enzymatic Substrate Coliform Test
*Escherichia coli*	SMEWW 9223 B
pH	SMEWW4500H+B—Eletrometric Methods
Turbidity	SMEWW 2130 B. Nephelometric Method

**Table 2:** The obtained values of the first shell, coordination number (CN), bond length(R) and Debye–Waller *σ*^2^ factor for nearest neighbor (Ag–Ag), (Zn–O), and (Zn–Zn) scattering from EXAFS fitting. The number in the parenthesis indicates the uncertainty in the last digit.

Sample	Shell	^C^N (1st Shell)	R (Å)	$\sigma_{Ag-Ag}^{2}\left({Å}^{2}\right)\;$	$\sigma_{Zn-O}^{2}\left({Å}^{2}\right)\;$	$\sigma_{Zn-Zn}^{2}\left({Å}^{2}\right)\;$
Ag/ ZnO (I)	Ag-Ag	11.9 (0)	2.87 (0)	0.008 (0)	-	-
	Zn–O	2.9 (4)	1.96 (0)	-	0.004 (2)	-
	Zn–Zn	11.9 (0)	3.22 (0)	-	-	0.01 (0)
Ag/ ZnO (II)	Ag-Ag	8.9 (0)	2.86 (0)	0.009 (0)	-	-
	Zn–O	2.9 (3)	1.96 (0)	-	0.003 (1)	-
	Zn–Zn	11.9 (0)	3.23 (3)	-	-	0.01 (0)

**Table 3:** Results validated according to the Standard Methods For The Examination of Water and Wastewater—23th Edition—SMEWW.

Sample	Parameters			
	Total Coliforms (MPN/100 mL)	*Escherichia coli* (MPN/100 mL)	pH	Turbidity (NTU)
Contaminated Water	>2419.6	59.4	7.5	28.1
Pure ZnO	Presence	Presence	8.2	1.5
Ag/ZnO (I)	<1 (Absence)	<1 (Absence)	7.7	1.9
Ag/ZnO (II)	<1 (Absence)	<1 (Absence)	7.5	3.1

**Table 4:** Relative concentration of elements evaluated by XPS.

Sample	Elements	Atomic Concentration (%)	
		Before	After
Ag/ZnO (I)	C1s	5.6	8.2
	O1s	46.0	43.0
	Zn 2p_3_	47.4	47.8
	Ag 3d	1.0	1.0
Ag/ZnO (II)	C1s	11.9	10.3
	O1s	43.0	45.0
	Zn 2p_3_	43.3	43.0
	Ag 3d	1.8	1.7

## Data Availability

Not applicable.

## Supplementary Materials

The following supporting information can be downloaded at: https://www.mdpi.com/article/10.3390/nano12101764/s1. Figure S1: BSE-SEM image of Ag/ZnO (I) (a), enlarged area of line analysis (b) and SEM-EDX line scan profiles of Zn (magenta), O (green) and Ag (orange) (c). Orange line in image a indicate the area of line analysis. Figure S2: BSE-SEM image of Ag/ZnO (II) with EDXS elemental maps of Zn, O and Ag. Scale bar on the elemental maps is 250 nm. Orange frame in BSE-SEM image indicates area of analysis. Figure S3: SEM images of Ag/ZnO (I) (a) and Ag/ZnO (II) (b) captured with a backscattered electron (BSE) detector. Figure S4: Characteristic TEM images of Ag-loaded ZnO sample prepared by ex situ doping (Ag/ZnO (II)). The white rectangular frame in the image a indicates area of (b). The round particle in (b) is crystalline. The average d-spacing is 0.25 nm and corresponds to (004) planes of hexagonal Ag (ICSD No. 01-870-0598).
