# Supplementary material for: Synthesis and Characterization of Ag/ZnO Nanoparticles for Bacteria Disinfection in Water
Source: Nanomaterials (Basel). 2022 May 22;12(10):1764. doi: 10.3390/nano12101764 (PMC9145672; doi:10.3390/nano12101764)
Supplement: Supplementary file 1 [file nanomaterials-12-01764-s001.zip › nanomaterials-1691698-supplementary.pdf]

nanomaterials

Article

Synthesis and Characterization of Ag/ZnO Nanoparticles for Bacteria Disinfection in Water

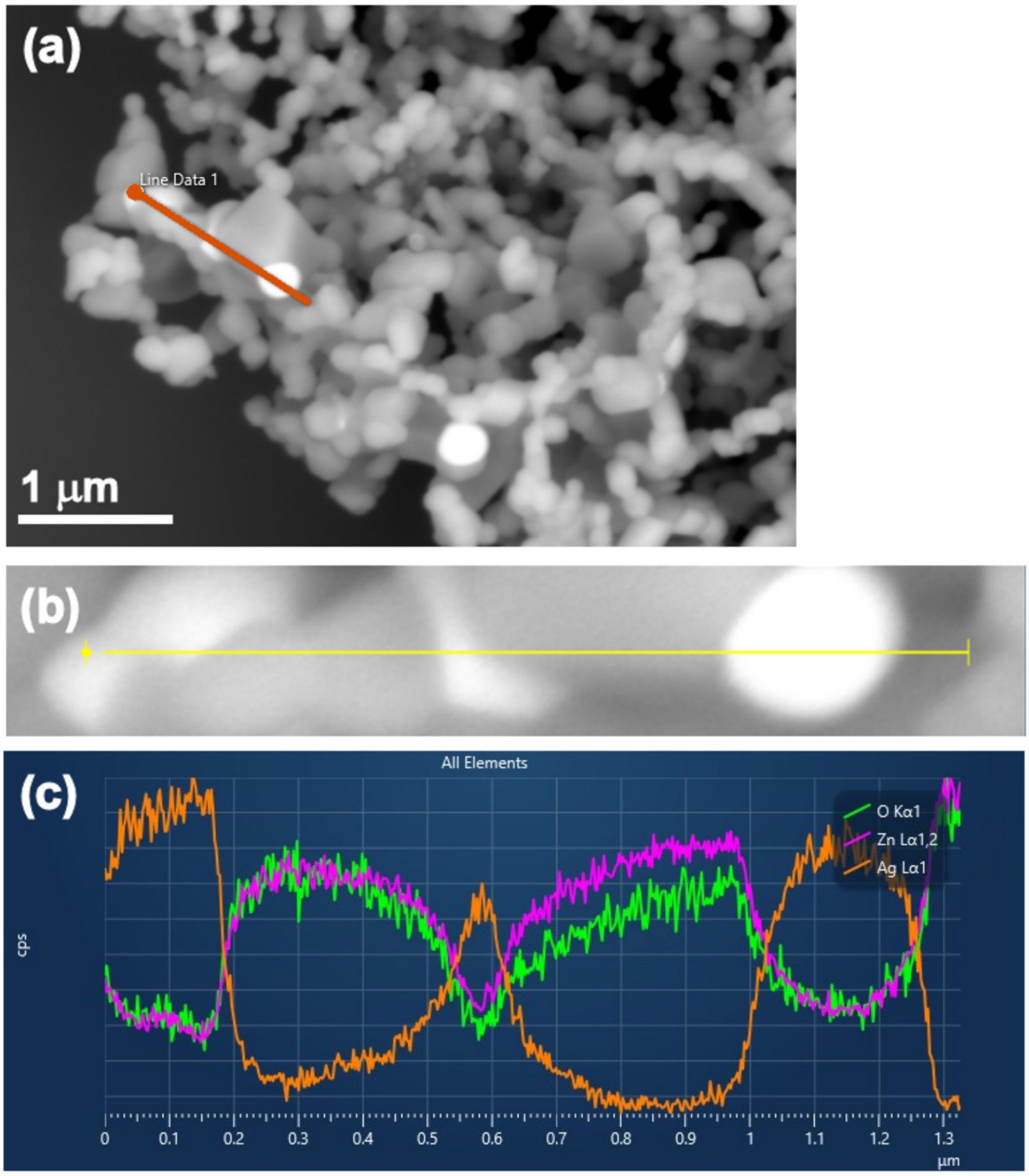

Figure S1. BSE-SEM image of Ag/ZnO (I) (**a**), enlarged area of line analysis (**b**) and SEM-EDX line scan profiles of Zn (magenta), O (green) and Ag (orange) **(c).** Orange line in image a indicate the area of line analysis.

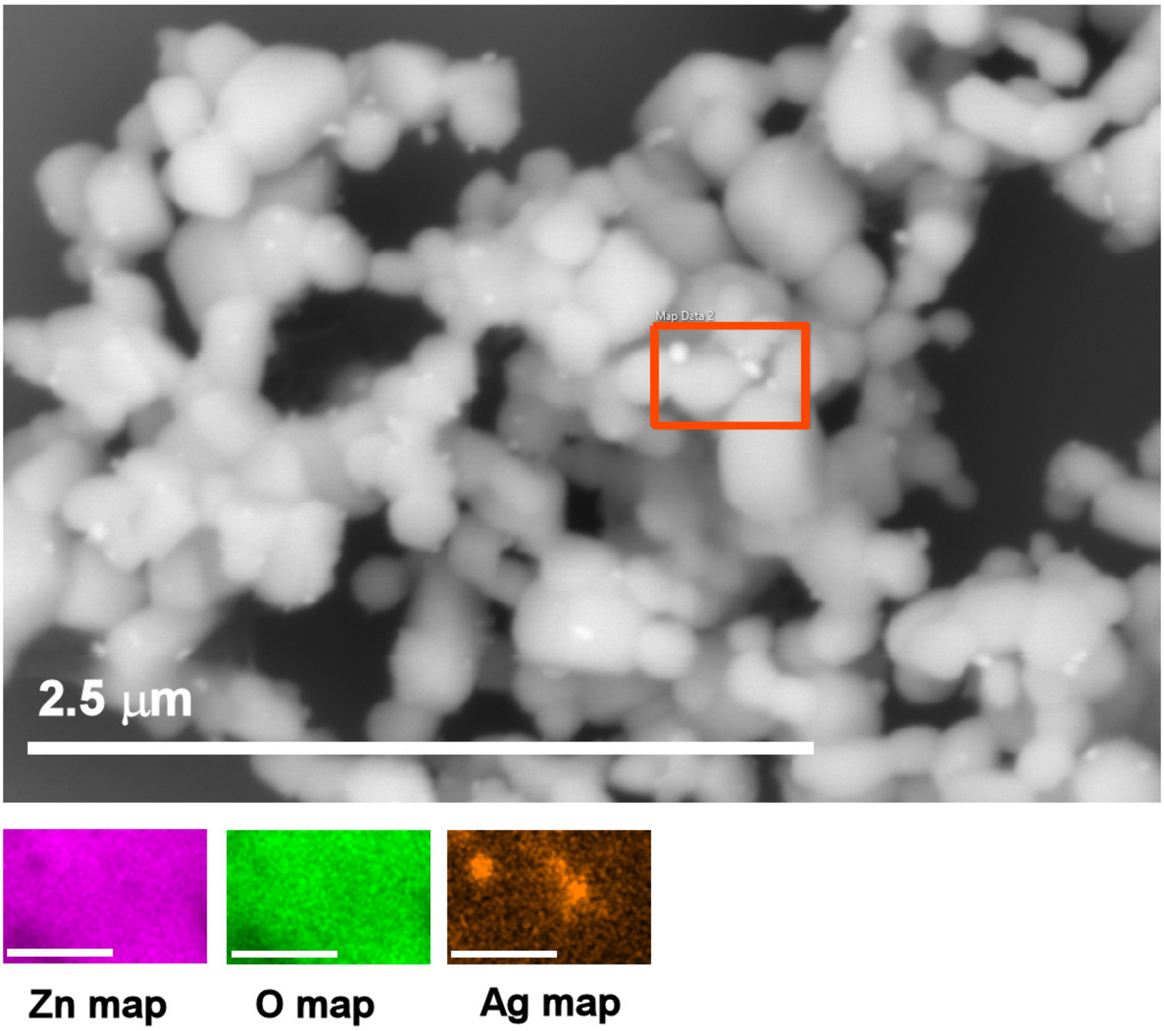

Figure S2. BSE-SEM image of Ag/ZnO (II) with EDXS elemental maps of Zn, O and Ag. Scale bar on the elemental maps is 250 nm. Orange frame in BSE-SEM image indicates area of analysis.

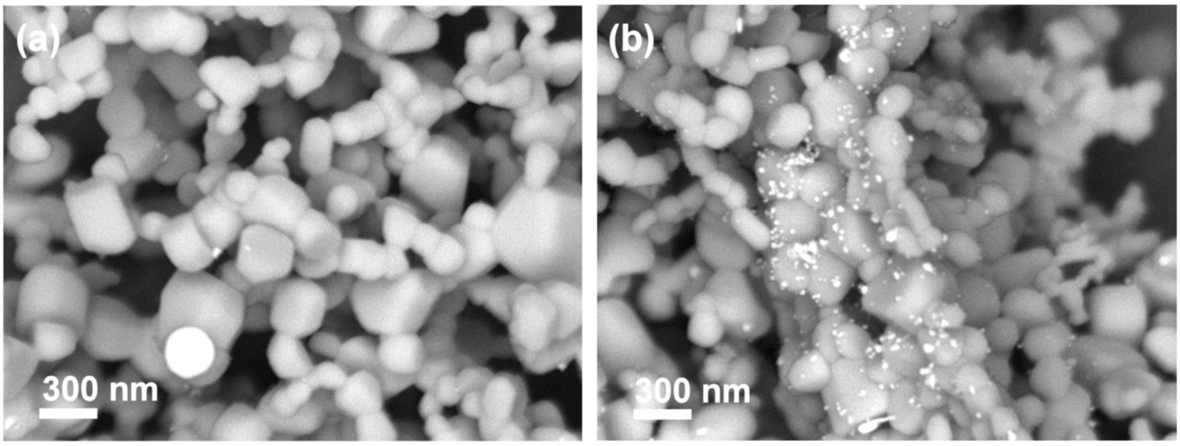

Figure S3. SEM images of Ag/ZnO (I) (**a**) and Ag/ZnO (II) (**b**) captured with a backscattered electron (BSE) detector.

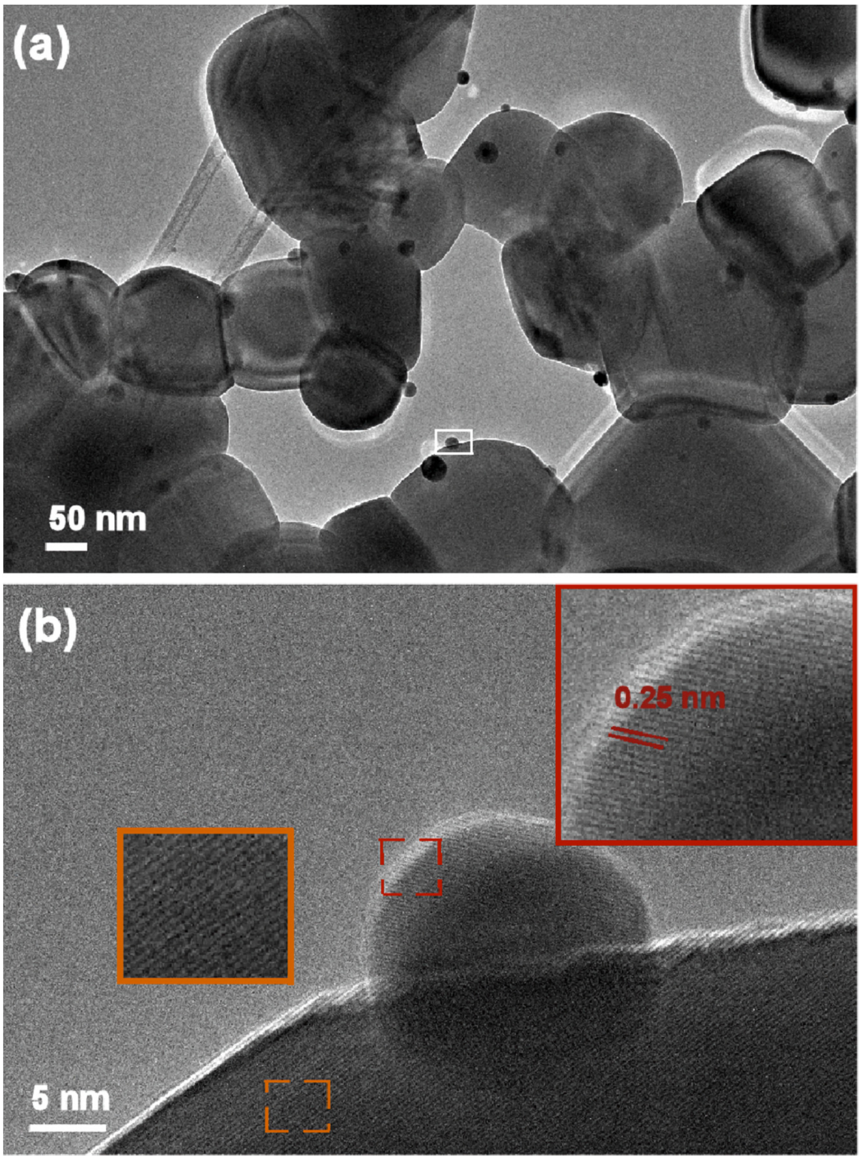

Figure S4. Characteristic TEM images of Ag loaded ZnO sample prepared by ex situ doping (Ag/ZnO (II)) The white rectangular frame in the image (**a**) indicates an area of the image (**b**). The red and orange frames indicate the areas of the HRTEM insets in the image b. The round particle in the image b is crystalline, the average d-spacing is 0.25 nm and corresponds to (004) planes of hexagonal Ag (PDF No. 01-087-0598).
